# A Future for Autologous Hematopoietic Stem Cell Transplantation in Type 1 Diabetes

**DOI:** 10.3389/fimmu.2018.00690

**Published:** 2018-04-06

**Authors:** Kayleigh M. van Megen, Ernst-Jan T. van ’t Wout, Stephen J. Forman, Bart O. Roep

**Affiliations:** ^1^Department of Diabetes Immunology, Diabetes & Metabolism Research Institute, Beckman Research Institute at the City of Hope, Duarte, CA, United States; ^2^Department of Hematology & Hematopoietic Cell Transplantation, T Cell Therapeutics Research Laboratory, City of Hope Beckman Research Institute and Medical Center, Duarte, CA, United States; ^3^Department of Immunohaematology and Blood Transfusion, Leiden University Medical Center, Leiden, Netherlands

**Keywords:** autologous hematopoietic stem cell transplantation, type 1 diabetes, immunotherapy, immune regulation, beta cells, personalized therapy, insulin independence

## Introduction

Type 1 diabetes (T1D) is an autoimmune disease caused by destruction of insulin producing β-cells in the pancreas. Standard of care therapy consists of life long symptomatic insulin treatment and in rare and severe cases patients undergo islet transplantation (1). Until today, autologous hematopoietic stem cell transplantation (aHSCT) proved to be the only intervention therapy for T1D reaching complete and sometimes even lasting remission (2–7). In spite of many other immunotherapies assessed around the globe, none matched the clinical efficacy of aHSCT (8, 9). Indeed, aHSCT had insulin-independency as primary end-point, rather than delayed loss of insulin production or decreased insulin needs. aHSCT is already widely and successfully used as a treatment for hematological malignancies (10, 11). Interestingly, one diabetic patient, when treated with aHSCT for multiple myeloma, became insulin independent (12). aHSCT was evaluated as a treatment for several autoimmune disorders as well, such as rheumatoid arthritis (13), systemic sclerosis (14, 15), multiple sclerosis (16), and juvenile idiopathic arthritis (17). By 2012, up to 3,000 aHSCT had been performed for autoimmune diseases (18). Yet, in the case of T1D, aHSCT remains controversial (19–21).

Indeed, the use of aHSCT as a strategy to cure T1D has been received with mixed enthusiasm. Concerns were raised about the short follow-up, the possibility that a positive effect of aHSCT may be attributable to a honeymoon phase and the absence of a placebo-treated trial arm for comparison (19, 21). Furthermore, the ethics of including minors in the trial was being questioned (19). Although valid at the time, these concerns have all since been addressed, as will become evident in the following paragraphs.

## aHSCT in T1D

The rationale behind using aHSCT in autoimmune diseases is to halt autoimmune destruction of the targeted tissue and reestablish tolerance. While the mechanism by which this is achieved remains incompletely resolved, the importance of a diverse T-cell receptor repertoire (22), thymus reactivation (23), and the number of regulatory T-cells (Treg) has been established (24).

The first evidence to demonstrate that aHSCT can reestablish tolerance in new-onset T1D patients comes from Voltarelli et al. (25, 26). Recent-onset (<6 weeks) T1D patients were included to undergo aHSCT with mobilized [cyclophosphamide (2.0 g/m^2^) and granulocyte colony-stimulating factor (10 µg/kg/day)] peripheral blood-derived hematopoietic stem cells after an intermediate-intensity conditioning regimen consisting of cyclophosphamide (200 mg/kg total) and rabbit antithymocyte globulin (4.5 mg/kg total). Similar mobilization and conditioning regimes were used in other discussed studies, unless mentioned otherwise. In total, 25 patients were included, of which 21 were treated according to protocol and became insulin independent, for a median of 43 months (2); a result unmatched by any intervention therapy up until this point. These results were substantiated independently around the world, accomplishing insulin independence in all studies, with maximum insulin independence ranging from 38 to 56 months and increasing with further follow-up (3–7). These studies prove that aHSCT is a promising therapy for T1D, while providing crucial and unique metabolic and immunological data of T1D patients in remission (27, 28).

## Balancing the Risk of aHSCT with the Risk of Diabetes-Associated Complications

Depending on the intensity of the conditioning regime, aHSCT can cause a wide range of complications. In the T1D trials (2–7), these ranged from relatively mild symptoms such as febrile neutropenia, nausea, and alopecia to more severe complications such as *de novo* autoimmunity and systemic infections, which in one case resulted in an unfortunate death (7). Temporal oligospermia was witnessed in some of the studies, but not all. Of note, multiple children have been conceived after aHSCT. Apart from these complications, there is also a concern of increased risk of malignancies after aHSCT, particularly myelodysplasia. With allotransplantation, this risk is well established and can be attributed to the heavy conditioning regime, while this regime is much milder in the autologous setting for autoimmune diseases. Furthermore, in contrast to aHSCT as a treatment for malignancies, stem cells of T1D patients have not sustained any damage from previous chemotherapy. Consequently, the incidence of malignancies was reported to be lower, although further prospective studies with longer follow-up and proper control groups are warranted to asses if these malignancies are aHSCT related (29).

Containment of adverse events from aHSCT is constantly improving as illustrated by decreased morbidity and mortality to <1% (30). Furthermore, in the setting of T1D, it will be performed in relatively young and otherwise fit subsets of patients with a low to intermediate conditioning regimen (2, 31), associated with reduced risk (29) without compromising treatment efficacy. This was attested by a recent trial exploring the possibility of a simplified method of aHSCT in an outpatient setting, with a conditioning regime consisting of cyclophosphamide (2.0 g/m^2^ total) and fludarabine (120 mg/m^2^ total), still reaching 44% prolonged insulin independence for up to 56 months and beyond, without significant adverse effects (4).

To make a compelling and fair case of aHSCT in T1D, the complications of aHSCT need to be juxtaposed with the short- and long-term complications of T1D. It is important to realize that acute and possibly life threatening events related to T1D and insulin treatment such as a hypoglycemic coma (32) and diabetic ketoacidosis (DKA) (33) are not uncommon. Indeed, T1D remains a deadly disease, where insulin therapy merely provides palliative care. In addition to a significantly reduced life expectancy, T1D also imposes severe and often lifelong negative impact on the quality of life of T1D patients. The major burden of the disease is caused by long-term micro- and macrovascular complications, with T1D still being a main cause of end stage renal disease and non-inherited blindness (34, 35). Even with optimal education and state-of-the-art treatment options, good glycemic control is not achieved in the vast majority of patients (36). This is of particular importance, since good glycemic control early in the course of the disease reduces long-term complications and preserves endogenous insulin production (37). Interestingly, patients that experienced a honeymoon phase showed significantly less macrovascular complications after 7 years of follow-up (38, 39). This could imply that a similar effect can be expected from an aHSCT induced prolonged period of insulin independence.

Importantly, side effects are inherent to immunotherapy. The adverse events of, for instance, DMARD, TNF blockers, sirolimus, cyclosporine, azathioprine, prednisone, thymoglobulin, alemtuzumab, or imatinib, all considered in the context of T1D, are certainly not negligible.

## Clinical Outcome of aHSCT Corresponds with the Degree of islet Autoreactivity before Therapy

Currently, after almost 15 years of experience in the application of aHSCT for the treatment of T1D, much knowledge has been gained about the mechanism of action of aHSCT and, concomitantly, about which patient population benefits most (2, 3, 5–7, 27, 28, 40–42).

Earlier this year, the first aHSCT in T1D trial reported its *ad hoc* analysis with a mean follow-up of 67.5 months (some patients remain insulin-independent beyond 106 months) and included 25 patients (2). HLA-A2 positive patients were divided into low and high cytotoxic T lymphocytes (CTL) autoreactivity groups according to the cumulative frequencies of islet-specific CTLs at baseline. Low CTL autoreactivity associated with higher c-peptide levels after aHSCT compared with high CTL autoreactivity. Furthermore, while 83% of patients in the high CTL group had resumed insulin therapy at 24 months after aHSCT, all patients with low frequencies of islet-autoreactive CTLs at baseline remained insulin independent. In addition, patients were divided into those with “short-remission” and “prolonged remission” depending on whether they were insulin-free for less or more than 3.5 years after aHSCT, respectively. A trend was seen of persistently lower cumulative frequencies of islet-specific CTLs in the prolonged remission group compared with the short-remission group. This outcome may point that the conditioning regimen with thymoglobulin was insufficient to deplete autoreactive T-cells. Diabetes relapse could then result from clonal expansion of autoreactive CTLs that escaped the conditioning procedure. In any case, these immunological parameters associated with superior or inferior clinical outcome of aHSCT before therapy point to patient and disease heterogeneity and present a good case for personalized and precision medicine in which tailoring the conditioning therapy might lead to more effective reversal of islet autoimmunity.

Additional evidence in favor of an immunogenic heterogeneity that relates to the outcome of aHSCT came from a study of 13 patients that was conducted in China with a mean follow-up of 42 months (5). Expressing more than one preexisting autoantibody negatively correlated with the preservation of beta-cell function as quantified by c-peptide levels. Yet, a larger study including 123 patients with a mean follow-up of 16 months found no difference in baseline presence of any of the autoantibodies between responding and non-responding patients (27). Serum levels of interleukin-10, interleukin-4, transforming growth factor-β, and fasting c-peptide after aHSCT correlated with the number of infused CD34+ cells, whereas tumor-necrosis factor-α (TNF-α) and insulin doses showed an inverse relation. Furthermore, prolonged insulin-free survival was negatively correlated with baseline TNF-α levels, which may provide another suitable negative predictor of prolonged remission (3).

In summary, current clinical evidence points to heterogeneity between patients and in disease, as well as provides immune correlates of disease remission or relapse that may offer opportunity for patient selection, precision medicine, and guidance for tailored immunotherapy following aHSCT.

## The Success of aHSCT in Relation to Preexisting Functional Beta-Cell Mass

Besides a baseline immune signature, *post hoc* analyses have revealed the importance of preexisting beta-cell mass for the outcome of aHSCT (27). One small study (5) found that the baseline c-peptide level was a positive predictor of post-aHSCT c-peptide levels, which was corroborated by other, larger studies (3). The largest study including 123 patients stratified subjects into a responder group and a non-responder group according to the presence of a post-aHSCT clinical response assessed by a β-score (27). The β-score is mainly used in the islet transplantation setting and consists of four components: fasting plasma glucose, HbA1c, c-peptide, daily insulin use or usage of oral hypoglycemic agents. The β-score was already significantly higher at baseline in responders compared with non-responders. Moreover, baseline fasting c-peptide levels proved to be an effective positive predictor of prolonged remission and the age of onset of diabetes a negative predictor. Obviously, baseline c-peptide levels are an indication of functional β-cell mass (27), although increasing evidence points to a disconnect between beta-cell mass and function in the case of diabetes (43, 44). β-Cell regeneration may occur until adolescence, after which regenerative capacity appears to stagnate (45). Indeed, early intervention within 6 weeks after diagnosis of T1D led to remission in the vast majority of cases, whereas later intervention achieved remission in less than half of the cases (42), suggesting that timely therapy matters.

The influence of DKA before aHSCT on clinical outcome could be substantial (6). Indeed, DKA at diagnosis has been associated with lower c-peptide levels, higher insulin needs and HbA1c levels, suggesting lower remaining β-cell function (46). Yet, another trial including 24 patients with 52 months as a mean follow-up found no relation between duration of insulin independence and the time from diagnosis to AHSCT, baseline c-peptide levels, nor number of CD34+ cells (7).

To summarize, patients with sufficient beta-cell function at baseline, no DKA at diagnosis, and treated early after diagnosis appear to benefit most. These characteristics all point toward the pivotal role of remaining functional beta-cell mass for success of aHSCT in T1D (27). To verify whether the age of onset matters (3), inclusion of minors in trials of aHSCT in T1D would be required. The potential capacity to regenerate their beta cells would further support considering young patients to offer this intervention therapy. Teenagers are a particularly challenging population to treat as diabetes-related distress, which is present in one-third of adolescents with T1D, is linked to poor glycemic control (47–49). Consequently, 84% of teens do not reach target HbA1c levels (36), which jeopardizes their future health with regards to increased long-term complications, but also their career perspectives (50).

## Selecting Eligible Patients for aHSCT in T1D

**Figure 1:**
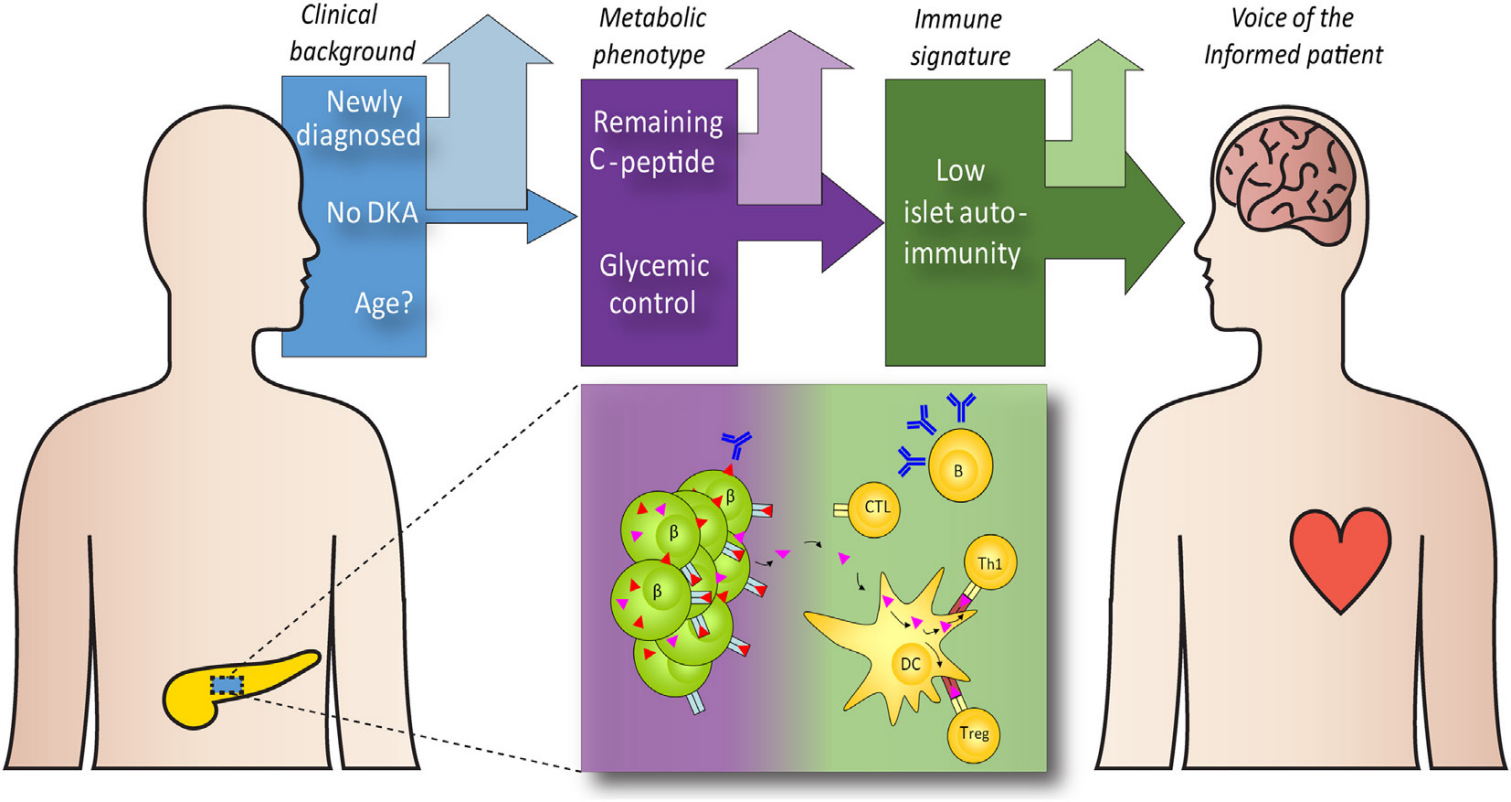
Guidance on the selection of type 1 diabetes (T1D) patients for autologous hematopoietic stem cell transplantation (aHSCT). aHSCT is unlikely to benefit all T1D patients. Factors that may help selecting the preferred candidates include the clinical background [disease duration, age, and diabetic complications, such as diabetic ketoacidosis (DKA)], metabolic features [remaining functional beta-cell mass (β), glycemic control, HbA1c] and immunopathogenic features [the number and type of islet autoantibodies, the frequency and specificity of islet-autoreactive cytotoxic T lymphocytes (CTL), and other effector (Th1) and regulatory (Treg) immune cells, and cytokine profiles]. With the opportunity to identify patient subgroups with particularly great or smaller chances for clinical benefit, we propose that we engage the patient community to guide shared decision-making.

Understanding which patient groups respond better to aHSCT and why, enables us to transform aHSCT from a general therapy to personalized medicine, thus envisioning a future of aHSCT in T1D. Yet, we contend that the choice for aHSCT as therapeutic option is not confined to the care providers. The voice of the patient is equally relevant, both in terms of refusing the risk for treatment related adverse events or accepting these in favor of temporal disease remission, preservation of beta-cell function, and reduced risk of diabetic complications. In case of minors, parents face the difficult task of weighing the best therapy for the patient in consultation with the care provider, which makes careful information provision even more important. We envision a future in which care providers, in dialog with the patient and caregivers, use a framework of evidence-based risk assessment to assess whether aHSCT is a viable option (see Figure 1).

## Conclusion

While aHSCT will not be the magic bullet universally curing T1D, there is a promising future for its implementation in a distinct group of patients (20). Indeed, none of the alternative intervention strategies match, or even get close to, the clinical outcome achieved in a considerable number of patients treated with aHSCT. We propose that this patient group should be identified, diligently informed and offered the possible benefits of an extended period of insulin-free and burden-free survival, while medical science continues their pursuit of developing alternative intervention strategies for those less eligible, or declining, aHSCT. T1D enters the era of personalized medicine.

## Author Contributions

KM and ET performed a literature review, contributed to the design of the work, analyzed the data, and wrote and edited the manuscript. KM designed the figure. BR conceived the idea of this work. BR and SF analyzed the data, revised and edited the manuscript, and contributed to the discussion. All the authors provided approval for publication and are accountable for all aspects of the work.

## Conflict of Interest Statement

The authors declare that the research was conducted in the absence of any commercial or financial relationships that could be construed as a potential conflict of interest.
